# A Machine Learning Approach to Predict the Rehabilitation Outcome in Convalescent COVID-19 Patients

**DOI:** 10.3390/jpm12030328

**Published:** 2022-02-22

**Authors:** Sarah Adamo, Pasquale Ambrosino, Carlo Ricciardi, Mariasofia Accardo, Marco Mosella, Mario Cesarelli, Giovanni d’Addio, Mauro Maniscalco

**Affiliations:** 1 Istituti Clinici Scientifici Maugeri IRCCS, Bioengineering Unit of Telese Terme Institute, 82037 Telese Terme, Italy; sarah.adamo@unina.it (S.A.); carloricciardi.93@gmail.com (C.R.); cesarell@unina.it (M.C.); gianni.daddio@icsmaugeri.it (G.d.); 2 Department of Information Technology and Electrical Engineering, University of Naples “Federico II”, 80125 Naples, Italy; 3 Istituti Clinici Scientifici Maugeri IRCCS, Cardiac Rehabilitation Unit of Telese Terme Institute, 82037 Telese Terme, Italy; pasquale.ambrosino@icsmaugeri.it; 4 Istituti Clinici Scientifici Maugeri IRCCS, Pulmonary Rehabilitation Unit of Telese Terme Institute, 82037 Telese Terme, Italy; mariasofia.accardo@icsmaugeri.it (M.A.); marco.mosella@icsmaugeri.it (M.M.)

**Keywords:** COVID-19, machine learning, exercise, rehabilitation, disability, occupational medicine, chronic disease, outcome

## Abstract

Background: After the acute disease, convalescent coronavirus disease 2019 (COVID-19) patients may experience several persistent manifestations that require multidisciplinary pulmonary rehabilitation (PR). By using a machine learning (ML) approach, we aimed to evaluate the clinical characteristics predicting the effectiveness of PR, expressed by an improved performance at the 6-min walking test (6MWT). Methods: Convalescent COVID-19 patients referring to a Pulmonary Rehabilitation Unit were consecutively screened. The 6MWT performance was partitioned into three classes, corresponding to different degrees of improvement (low, medium, and high) following PR. A multiclass supervised classification learning was performed with random forest (RF), adaptive boosting (ADA-B), and gradient boosting (GB), as well as tree-based and k-nearest neighbors (KNN) as instance-based algorithms. Results: To train and validate our model, we included 189 convalescent COVID-19 patients (74.1% males, mean age 59.7 years). RF obtained the best results in terms of accuracy (83.7%), sensitivity (84.0%), and area under the ROC curve (94.5%), while ADA-B reached the highest specificity (92.7%). Conclusions: Our model enables a good performance in predicting the rehabilitation outcome in convalescent COVID-19 patients.

## 1. Introduction

The coronavirus disease 2019 (COVID-19) is a syndrome with a number of clinical manifestations, ranging from mild symptoms to severe complications necessitating intensive care unit (ICU) admittance [1]. After the acute disease, convalescent COVID-19 patients may experience several persistent symptoms, such as fatigue and muscular weakness [2], with a residual pulmonary impairment potentially lasting for months after a negative swab test [3]. Overall, given the high proportion of patients with such persistent manifestations, the new paradigm of a “post-acute COVID-19 syndrome” has been introduced [3]. Thus, the need for an early and multidisciplinary rehabilitation has been proposed [4,5,6,7]. Unfortunately, the information on the effectiveness of this approach in the post-acute care setting is still to be determined, given the absence of a general consensus on the rehabilitation programs and the lack of adequate prediction tools [8].

Among the functional outcome measures of pulmonary rehabilitation (PR), the 6-min walking test (6MWT) is widely accepted as an accurate and cost-effective method [9]. 6MWT is commonly used to measure physical activity and exercise capacity, correlating with both peak oxygen consumption and handgrip strength [10].

In the last years, the machine learning (ML) approach has been increasingly used, allowing researchers to implement algorithms that analyze datasets in order to predict the onset of a disease. Moreover, ML algorithms have been successfully used to predict rehabilitation outcomes in neurology [11], orthopaedics [12], and cardiology [13]. Recently, ML has also been used to find hidden patterns among patients affected by COVID-19, employing features extracted from X-ray and computed tomography (CT) images with good results [14].

While ML has been extensively employed as a means of triaging COVID-19 patients during the acute phase [15], no studies have used this approach to classify the factors influencing the rehabilitative outcome in the post-acute care setting.

Using the clinical characteristics of convalescent COVID-19 patients hospitalized for PR, the aim of our study was to develop a model predicting the effectiveness of multidisciplinary rehabilitation in terms of improved performance at the 6MWT. 

## 2. Materials and Methods

### 2.1. Study Population

Convalescent COVID-19 patients referring to the Pulmonary Rehabilitation Unit of Istituti Clinici Scientifici Maugeri Spa SB, IRCCS of Telese Terme, Benevento, Italy, were consecutively evaluated for enrolment. Inclusion criteria were recent severe acute respiratory syndrome coronavirus 2 (SARS-CoV-2) infection, as confirmed by reverse transcription polymerase chain reaction (RT-PCR); severe-to-critical COVID-19 according to World Health Organization (WHO); negative nasopharyngeal swab for SARS-CoV-2 in the past 2 months; and indication for in-hospital PR due to persistent clinical manifestations of COVID-19 after the acute phase. Exclusion criteria were age < 18 years and inability to understand the informed consent or poor compliance with the study procedures in the investigator’s opinion. Patients with missing data for the outcome of interest were excluded from the study.

Whenever appropriate and applicable, this study followed the Strengthening the Reporting of Observational Studies in Epidemiology (STROBE) reporting guidelines [16]. The protocol was approved by the Institutional Review Board of “Istituto Nazionale Tumori, Fondazione Pascale”, Naples, Italy, with reference number ICS 11/20, and all patients provided written informed consent to use their de-identified data.

### 2.2. Data Collection and Analysis

After informed consent signature, the main demographic and clinical characteristics were collected in all included patients. All study procedures were performed at baseline and after conclusion of the PR program.

A blood gas analyzer (ABL 825^®^ FLEX BGA, Radiometer Medical Aps, Copenhagen, Denmark) was used to measure arterial oxygen (PaO_2_) and carbon dioxide tension (PaCO_2_). Spirometry parameters, lung volumes, and diffusion capacity for carbon monoxide (DLCO) were measured by using automated equipment (Vmax^®^ Encore, Vyasis Healthcare, Milan, Italy) according to American Thoracic Society/European Respiratory Society (ATS/ERS) guidelines [17,18]. Forced expiratory volume in 1 s (FEV_1_), forced vital capacity (FVC), and DLCO were expressed both in liters (L) and percent of predicted values (FEV_1_%, FVC%, and DLCO%, respectively).

The Barthel score and the COPD Assessment Test (CAT) were calculated to determine the level of functioning and to monitor improvements in activities of daily living over time [19,20]. The 6MWT was also performed in accordance with the ATS/ERS guidelines [21]. The 6-min walking distance (6MWD) was reported in meters.

Since the 6MWD parameter is a good outcome measure in rehabilitation [22], a normalization was performed in order to obtain a class column to conduct ML analysis. For this purpose, all 6MWD values before and after rehabilitation were normalized in percentage depending on the theoretical maximum for each patient, as determined according to the ATS guidelines for the 6MWT [14] and considering age, sex, and body mass index (BMI), as follows:

(1) $${6\mathrm{MWD}}_{\mathrm{Maximum}\%\_\mathrm{before}}\;=\;\frac{6\mathrm{MWD}\_\mathrm{before}}{6\mathrm{MWD}\_\mathrm{Maximum}}\times 100$$

(2) $${6\mathrm{MWD}}_{\mathrm{Maximum}\%\_\mathrm{after}}\;=\;\frac{6\mathrm{MWD}\_\mathrm{after}}{6\mathrm{MWD}\_\mathrm{Maximum}}\times 100$$

(3) $$6\mathrm{MWD}\;=\;6{\mathrm{MWD}}_{\mathrm{Maximum}\%\mathrm{after}}-6{\mathrm{MWD}}_{\mathrm{Maximum}\%\mathrm{before}}$$

Consequently, Δ6MWD has been partitioned into three classes corresponding to different degrees of improvement following rehabilitation:

- Class 0: low improvement, between 0 and 20%;
- Class 1: medium improvement, between 20 and 40%;
- Class 2: high improvement, over 40%.

The IBM SPSS Statistics V. 27.0 system (Chicago, IL, USA) was used to compare demographic and clinical features of patients before and after rehabilitation through a univariate statistical analysis. The normality distribution of the data was assessed with the Kolmogorov–Smirnov test. Then a *t*-test for paired samples was performed for normally distributed data; otherwise, the Wilcoxon signed-rank test for paired samples was performed.

### 2.3. Pulmonary Rehabilitation Program

All enrolled patients underwent a 5-week PR program with daily sessions (6 sessions/week). Thus, a total of 30 sessions were planned, according to the official ATS/ERS guidelines [23]. PR consisted of physical exercise training, dietary counselling, and psychosocial counselling. Physical exercise training was the key point of the program, consisting of exercises to strengthen groups of muscles, treadmill walking, and stationary cycling. Lower- and upper-limb strengthening exercises were performed by using body and fixed weights at a load that could be supported for 8 to 10 repetitions before muscle exhaustion. Loads were increased when patients were able to complete 3 sets of 8–10 repetitions in two consecutive training sessions. Arm ergometry was planned for a 10 min/session at an intensity of 3 or 4 on the Rating of Perceived Exertion (RPE) 0 to 10 scale [24]. Treadmill walking duration was 15 min at PR initiation and was progressed to 30 min during the first 2 weeks, reaching an RPE score of 3 to 4. The intensity of lower-limb cycling intensity was set at an intensity aimed at scoring dyspnea or perceived exertion from 3 to 4 on the modified 0-to-10 category-ratio scale [24,25]. Patients also underwent flexibility and stretching exercises. A physiotherapist monitored and supervised participation.

### 2.4. Machine Learning Workflow

ML algorithms were implemented through KNIME analytics platform (v. 4.2.1), already successfully used in other biomedical studies [11,26,27]. In this study, a multiclass supervised classification learning was performed with tree-based and instance-based algorithms.

Overall, 189 instances were recorded, and a set of 30 features was chosen for modelling. Among them, 19 were continuous attributes and did not require a discretization, since they represented numerical clinical variables, and 11 were nominal attributes that were transformed in binary variables. A previous preprocessing was performed to replace missing values with rounded mean or most frequent value in numerical and categorical features, respectively. Then the Synthetic Minority Oversampling Technique (SMOTE) was applied in order to reduce data imbalance between classes: this technique oversamples minority classes by introducing synthetic samples along lines that join k-nearest neighbors of the same class [28]. Regardless, synthetic sample size was less than 50% of the entire dataset. The holdout method (70% training and 30% test) was used to train and validate random forest (RF), adaptive boosting (ADA-B), and gradient boosting (GB), as well as tree-based and k-nearest neighbors (KNN) as instance-based algorithms. Tree-based learning algorithms employ the decision-tree classifier, sorting instances down the tree from the root to some leaf nodes. These algorithms are a good method for discrete-valued target problems. Decision-tree learning methods usually perform well when instances are represented by attribute–value pairs and are robust to missing values and errors that could be contained in the training data. For the explained reasons, tree-based algorithms are generally better suited to medical and clinical issues where a classification is required. RF in an ensemble learning algorithm based on the bagging technique, which combines predictions of several trained models and returns the most voted result as an output. Another key concept of RF is randomization: each tree is trained on different and random sets of data and subsets of features [29]. ADA-B and GB are two ensemble and iterative learning algorithms that use the boosting principle. The former starts from a set of weak learners, usually decision stumps, and for each cycle, it assigns different weights to incorrect classifications, building a strong learner [30]. The latter optimizes weak learners results according to the gradient descent criterion: each single model is trained by minimizing the cost function, which, in this case, is the mean square error. Differently, instance-based algorithms use instances to perform classification tasks, assuming that similar instances have similar classifications, thus considering the most similar neighbors in terms of variables and attributes. Therefore, this method can be employed to explore a medical issue and to evaluate if different phenotypes depending on their characteristics can be identified. KNN is an instance-based model and one of the simplest classification algorithms that identifies similarity between k-neighbor samples by measuring their distances and then defines groups of k-similar samples. In this study, a Distance-Weighted KNN was employed, so the contribution of each attribute was evaluated according to its distance to the query point, and closer neighbors were greater weighted.

Feature importance was computed with RF to identify the most relevant features in classification through Information Gain (IG). IG is an entropy-based feature evaluation method, which considers how much information a feature can provide and how much this feature can be used in the classification process in order to measure its importance. In RF, feature importance is estimated by looking at how much prediction error increases when data for a certain variable are permuted while the others are left unchanged [31]. Then the IG of all the features was normalized and transformed into percentage in order to express and compare the contribution of each feature to the prediction.

Finally, the algorithms’ performances were evaluated through the following metrics, based on true negatives (TN), true positives (TP), total sample (TOT), false negatives (FN), and false positives (FP):

(4) $$\mathrm{Accuracy}\;=\;\frac{\mathrm{TN}+\mathrm{TP}}{\mathrm{TOT}}$$

(5) $$\mathrm{Sensitivity}\;=\;\frac{\mathrm{TP}}{\mathrm{TP}+\mathrm{FN}}$$

(6) $$\mathrm{Specificity}\;=\;\frac{\mathrm{TN}}{\mathrm{TN}+\mathrm{FP}}$$

AUROC = area under the receiver operating characteristics (ROC) curve (sensitivity − specificity). (7)

## 3. Results

Among 197 patients screened for eligibility, three (1.5%) were ineligible for protocol adherence issues. A total of two (1.0%) out of the 194 eligible patients dropped out before completion of the project requirements, while three (1.5%) refused to sign the informed consent.

Therefore, the study population consisted of 189 convalescent COVID-19 patients (74.1% males, mean age 59.7 years). In Table 1, the baseline demographic and clinical characteristics pertaining to the acute phase of COVID-19 are reported.

As shown in Table 2, convalescent COVID-19 patients showed a significant improvement in the main pulmonary function parameters and exercise capacity after PR.

In detail, as compared to baseline, a significant increase in PaO_2_ was documented (*p* < 0.001). Moreover, an improvement in most spirometry parameters was reported at the end of the PR program, with FEV_1_% changing from 76.66% predicted ± 19.78 to 84.51% predicted ± 17.69 (*p* < 0.001) and FVC% from 74.34% predicted ± 19.82 to 81.73% predicted ± 16.77 (*p* < 0.001). Similarly, DLCO% and total lung capacity (TLC) significantly increased after PR, from 55.02% predicted ± 19.40 to 61.13% predicted ± 20.98 (*p* < 0.001), and from 4.58 L ± 1.35 to 5.82 L (*p* = 0.017), respectively. A significant and consistent improvement in exercise capacity was also documented at the end of the PR program, with 6MWD changing from 156.41 m ± 123.83 to 304.32 m ± 135.67 (*p* < 0.001). Finally, self-assessment measures of health status impairment (CAT) and functional limitation (Barthel score) also significantly improved after PR (*p*-value always < 0.05).

### Machine Learning Anlysis

The three classes of normalized Δ6MWD corresponding to different degrees of improvement after PR were the following:

- Class 0, low improvement: 64 patients;
- Class 1, medium improvement: 95 patients;
- Class 2, high improvement: 30 patients.

They were oversampled through SMOTE, for a total sample size of 285 patients.

The set of features was composed of the variables reported in Table 1 and Table 2 and were passed in input to ML algorithms. The evaluation metrics are summarized in Table 3 per each algorithm.

**Figure 1:**
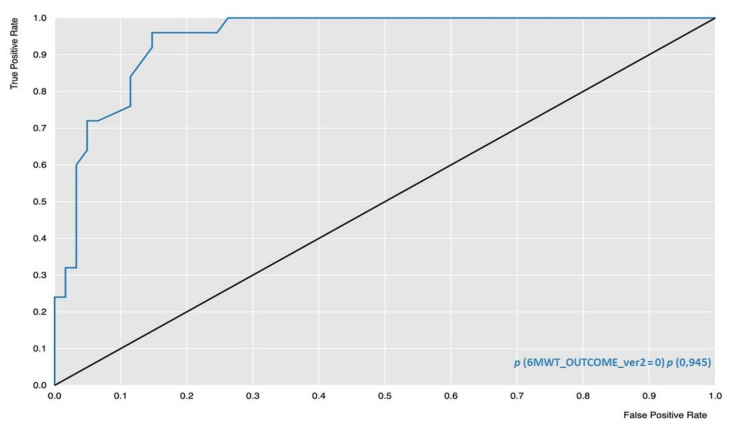
Receiver Operating Characteristic (ROC) curve of RF algorithm (blue line); ROC = 0.5, threshold for considering the model better than random guessing (black line).

RF obtained the best results in terms of accuracy (83.7%), sensitivity (84.0%), and AUROC (94.5%), while ADA-B reached the highest specificity (92.7%). Figure 1 shows the ROC curve of the RF algorithm, with Class 0 being the positive class value.

**Figure 2:**
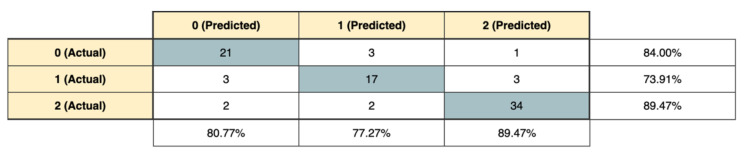
Confusion matrix of random forest (RF) algorithm.

Figure 2 shows the RF confusion matrix, which compares the predicted values and the actual values for each class, with the corresponding accuracies. Due to the well-balanced dataset, a high number of instances were correctly classified, as reported in the highlighted cells.

The 10 most important baseline features with the corresponding relative importance rankings are reported in Table 4.

Table 5 shows the distribution of these features among the three classes of improvement, with 6MWD, FEV_1_, FVC, FVC%, and PaO_2_ being significantly different between the three study groups (*p* always < 0.05).

## 4. Discussion

Based on an ML approach, the results of this study show the importance of some clinical and functional parameters in predicting the rehabilitation outcome in convalescent COVID-19 patients, expressed by an improved performance at 6MWT. Moreover, in line with previous evidence [32], our findings confirm the potential usefulness of multidisciplinary PR for COVID-19 patients in the post-acute care setting.

In this study, clinical and functional features of post-COVID-19 patients were explored through a univariate analysis and then employed as input for several ML algorithms in order to predict the percentage of improvement after rehabilitation. Our statistical analysis showed significant differences for the majority of functional and spirometry parameters before and after rehabilitation. The outcome measure identified for the ML analysis was the 6MWD, for which a normalization was performed. In detail, all the values were normalized depending on the theoretical maximum for each patient. Therefore, the evaluation was conducted on the basis of individual parameters and health status. After defining three ranges of improvement following rehabilitation, SMOTE was used to balance classes without altering the clinical significance of the dataset, and then ML algorithms were implemented.

Previous researchers focused on COVID-19 patients through a similar ML approach aimed at predicting mortality and stratifying risks correlated to comorbidities. For example, Gao et al. presented a prediction model trained and validated in over 2000 participants to stratify patients by mortality risk, using their clinical data on admission and obtaining an AUROC of 96.2% [33]. On the other hand, Hajifathalian et al. developed a prediction model to assess short-term mortality risk among hospitalized COVID-19 patients, based on patient age, hypoxia severity, mean arterial pressure, and presence of kidney disfunction. This model exhibited a similar performance in both internal (AUROC: 86.0%) and external validation (AUROC: 86.0%) [34]. Several other studies used ML to predict mortality [35] but also to evaluate the necessity of oxygen supplementation [36], to monitor pandemic-related psychopathology [37], to identify vaccine-related adverse events from Twitter data [38], and even to diagnose COVID-19 from cough audio signals [39]. However, to the best of our knowledge, no previous study used ML to predict the rehabilitation outcome in the post-acute phase of COVID-19.

Therefore, our study was the first specifically focusing on rehabilitation. The ML analysis was aimed at implementing algorithms that are able to predict clinical and functional improvements, overcoming good results in terms of accuracy (83.7%) and AUROC (94.5%). A relevant result of our model was the importance of certain spirometry and functional parameters as leading features in predicting the rehabilitative outcome in post-COVID-19 patients, expressed by an improved performance at the 6MWT. Beyond the baseline 6MWD, it is interesting to highlight the relevance of a number of pulmonary function parameters, including FEV_1_, FVC, DLCO, and TLC, potentially indicating restriction [40,41]. In detail, the strong interrelationship between FVC and lung volumes is one of the elements allowing us to clarify the nature of the lung damage, thus confirming the restrictive nature of the residual pulmonary involvement in our study population. Therefore, the fact that FVC and TLC are among the parameters that contribute the most to the prediction of the rehabilitation outcome suggests that, in line with previous evidence [32,42], there is a strong influence of the residual restrictive pattern on the disabling manifestations and the possibility of recovery after the acute phase. Accordingly, the role of DLCO as a main discriminating feature for the rehabilitation outcome emerges from our model, also suggesting the importance of interstitial or pulmonary vascular abnormalities in predicting the response to rehabilitation. Moreover, if the key prognostic role of some demographic variables is well established in COVID-19 [43], our prediction model further confirms the importance of age. Another noteworthy point is the presence of the CAT score as one of the features with greater relative importance in our model, as it suggests the key role of the self-assessment of health status improvement in predicting the rehabilitation outcome.

Of interest, although a significant difference between the three classes of improvement was observed for some—but not all—features, our findings indicate a better room for improvement among patients with greater functional limitations at baseline. This is partially in contrast with previous evidence on chronic obstructive pulmonary disease (COPD), showing that patients with COPD may benefit from PR regardless of disease severity [44]. In another study on 80 patients, in stark contrast to our results, 6MWD was directly correlated with the baseline FEV_1_, PaO_2_, and 6MWD [45]. Accordingly, Berry et al. previously documented an average increase in 6MWD after PR of 61.2, 72.7, and 34.2 m in mild, moderate, and severe COPD, respectively [46]. In our study on COVID-19 survivors, the fact that patients in group two presented a significantly lower functional status at baseline, as expressed by lower 6MWD and pulmonary parameters, indicates a negative association of most features chosen for modeling with the rehabilitation outcome. This apparently contrasting result may depend on the different nature of the disease, obstructive for COPD and mainly restrictive for COVID-19, on the different etiology, and, most important, on the different disease duration and course. While COPD is a chronic progressive disease, the current literature data suggest that most COVID-19 survivors may substantially improve their functional status [32,43], particularly following a rehabilitation program, with most patients showing no computed tomography abnormalities after 1 year from the acute phase [47], although this can often require a long time. This possibility of a consistent functional improvement may at least in part justify the lower functional status among patients with a higher degree of 6MWD improvement after PR.

Some potential limitations of our study should be addressed. Patients included in our protocol were all local residents from the Campania Region in Italy. This could somehow reduce the predictive value of our model, which would therefore need to be validated on other populations/ethnic groups. Moreover, the relatively low number of participants in our study suggests the need of further prospective studies on a larger sample in order to clarify which features chosen for modeling may have a positive or negative association with the rehabilitation outcome. Finally, since COVID-19 disease is proven to be a systematic disease that may also harm the cardiovascular and neurologic system, more clinical data from the cardiovascular, neurological, and myoskeletic system would help the assessment.

## 5. Conclusions

In addition to suggesting and confirming the favorable effect of rehabilitation on a range of functional parameters after the acute phase of COVID-19, our results support the importance of some clinical and demographic variables in predicting the rehabilitation outcome. Our model, despite needing further validation in larger external populations, could effectively assist clinicians in defining more personalized rehabilitation programs.

## Figures and Tables

**Table 1:** Baseline demographic and clinical characteristics of post-acute COVID-19 patients.

Patients, *N*	189
Age, years	59.7 ± 10.4
Female, *N*	49
Smokers, *N*	14
BMI, Kg/m^2^	29.1 ± 6.1
Hospitalization length, days	17.6 ± 15.2
Days from a negative swab	22.6 ± 17.8
High flow oxygen, *N*	42
Mechanical ventilation, *N*	47
Hypertension, *N*	86
Hypercholesterolemia, *N*	18
Hypertriglyceridemia, *N*	12
Diabetes, *N*	32
Heart failure, *N*	18
Atrial fibrillation, *N*	5
History of stroke/TIA, *N*	4

BMI, body mass index; TIA, transient ischemic attack.

**Table 2:** Main clinical features and pulmonary function tests before and after pulmonary rehabilitation (PR) in 189 post-acute COVID-19 patients.

	Before PR	After PR	*p*-Value
PaO_2_, mmHg	73.48 ± 14.98	80.91 ± 14.20	<0.001
PaCO_2_, mmHg	36.18 ± 5.37	36.94 ± 3.64	0.002
pH	7.45 ± 0.05	7.43 ± 0.04	<0.001
FEV_1_, L	2.34 ± 0.76	2.65 ± 0.75	<0.001
FEV_1_%, % predicted	76.66 ± 19.78	84.51 ± 17.69	<0.001
FVC, L	2.84 ± 0.96	3.19 ± 0.90	<0.001
FVC%, %predicted	74.34 ± 19.82	81.73 ± 16.77	<0.001
FEV_1_ / FVC	81.88 ± 9.70	81.15 ± 9.52	<0.001
RV, L	1.36 ± 0.73	1.43 ± 0.86	0.123
TLC, L	4.58 ± 1.35	5.82 ± 1.27	0.017
DLCO, mL/min/mmHg	10.71 ± 7.43	10.17 ± 8.15	0.002
DLCO%, % predicted	55.02 ± 19.40	61.13 ± 20.98	<0.001
6MWD, meters	156.41 ± 123.83	304.32 ± 135.67	<0.001
CAT	26.68 ± 3.25	9.51 ± 4.66	<0.001
Barthel	67.96 ± 29.68	94.34 ± 13.10	<0.001

PaO_2_, arterial oxygen tension; PaCO_2_, arterial carbon dioxide tension; pH, power of hydrogen; FEV_1_, forced expiratory volume in 1 s; FVC, forced vital capacity; RV, residual volume; TLC, total lung capacity; DLCO, diffusion lung of carbon monoxide; 6MWD, 6-min walk distance; CAT, COPD Assessment Test. Data are presented as mean ± standard deviation unless otherwise indicated.

**Table 3:** Evaluation metrics for each algorithm.

Algorithm	Accuracy	Sensitivity (%)	Specificity (%)	AUROC (%)
RF	83.7	84.0	91.8	94.5
ADA-B	81.4	71.0	92.7	88.5
GB	79.1	71.0	87.3	84.6
KNN	80.2	74.2	89.1	93.4

AUROC, area under the receiver operating characteristic curve; RF, random forest; ADA-B, adaptive boosting; GB, gradient boosting; KNN, k-nearest neighbors.

**Table 4:** Features information gain (IG) normalized and transformed into percentage for the 10 most important features chosen for modeling.

Feature	IG
6MWD, meters	10.62%
DLCO%, % predicted	6.25%
FVC, L	5.85%
DLCO, mL/min/mmHg	5.09%
FEV_1_, L	4.68%
PaO_2_, mmHg	4.67%
TLC, L	4.59%
CAT	4.57%
Age, years	4.53%
FVC%, % predicted	4.41%

6MWD, 6-min walking distance; DLCO, diffusing lung capacity for carbon monoxide; FVC, forced vital capacity; FEV_1_, forced expiratory volume in 1 s; PaO_2_, arterial oxygen tension; TLC, total lung capacity.

**Table 5:** Comparisons among the three classes of improvement following PR, according to the 10 most important features.

Features	Group 0 (*n* = 64)	Group 1 (*n* = 95)	Group 2 (*n* = 30)	*p*-Value
6MWD, meters	193.13 ± 131.77	171.20 ± 90.16	31.10 ± 56.69	<0.001
DLCO%, % predicted	55.70 ± 15.63	56.27 ± 12.82	49.97 ± 11.58	0.230
FVC, L	2.97 ± 0.62	2.92 ± 0.85	2.42 ± 0.65	0.001
DLCO, mL/min/mmHg	11.73 ± 6.77	10.97 ± 5.19	10.47 ± 4.11	0.682
FEV_1_, L	2.44 ± 0.52	2.34 ± 0.67	2.03 ± 0.58	0.003
PaO_2_, mmHg	75.19 ± 13.12	72.89 ± 12.33	65.03 ± 13.32	0.005
TLC, L	4.51 ± 0.85	4.71 ± 1.02	4.53 ± 1.11	0.736
CAT	26.92 ± 2.53	26.61 ± 1.36	27.00 ± 3.53	0.163
Age, years	62.56 ± 12.84	62.88 ± 8.62	63.97 ± 9.18	0.972
FVC%, % predicted	76.81 ± 15.56	76.57 ± 14.83	64.33 ± 14.22	<0.001

6MWD, 6-min walking distance; DLCO, diffusing lung capacity for carbon monoxide; FVC, forced vital capacity; FEV_1_, forced expiratory volume in 1 s; PaO_2_, arterial oxygen tension; TLC, total lung capacity.

## Data Availability

The data are available upon request to the corresponding author.
